# Sensitivity of a Simple Noninvasive Screening Algorithm for Chronic Thromboembolic Pulmonary Hypertension after Acute Pulmonary Embolism

**DOI:** 10.1055/s-0038-1636537

**Published:** 2018-02-27

**Authors:** Yvonne M. Ende-Verhaar, Dieuwertje Ruigrok, Harm Jan Bogaard, Menno V. Huisman, Lilian J. Meijboom, Anton Vonk Noordegraaf, Frederikus A. Klok

**Affiliations:** 1 Department of Thrombosis and Hemostasis, Leiden University Medical Center, Leiden, The Netherlands; 2 Department of Pulmonology, VU University Medical Center, Amsterdam, The Netherlands; 3 Department of Radiology and Nuclear Medicine, VU University Medical Center, Amsterdam, The Netherlands

**Keywords:** pulmonary embolism, CTEPH, screening algorithm, sensitivity, reproducibility

## Abstract

**Background** Recently, we constructed a noninvasive screening algorithm aiming at earlier chronic thromboembolic pulmonary hypertension (CTEPH) detection after acute pulmonary embolism (PE), consisting of a prediction score and combined electrocardiography (ECG)/N-terminal pro-brain natriuretic peptide (NT-proBNP) assessment. The aim of this study was to confirm the algorithm's sensitivity for CTEPH detection and to evaluate the reproducibility of its individual items.

**Methods** Two independent researchers calculated the prediction score in 54 consecutive patients with a history of acute PE and proven CTEPH based on clinical characteristics at PE diagnosis, and evaluated the ECG and NT-proBNP level assessed at the moment of CTEPH diagnosis. Interobserver agreement for the assessment of the prediction score, right-to-left ventricle (RV/LV) ratio measurement on computed tomography pulmonary angiography, as well as ECG reading was evaluated by calculating Cohen's kappa statistics.

**Results** Median time between PE diagnosis and presentation with CTEPH was 9 months (interquartile range: 5–15). The sensitivity of the algorithm was found to be 91% (95% confidence interval [CI]: 79–97%), indicating that 27 of 30 cases of CTEPH would have been detected when applying the screening algorithm to 1,000 random PE survivors with a 3% CTEPH incidence (projected negative predictive value: 99.7%; 95% CI: 99.1–99.9%). The interobserver agreement for calculating the prediction score, RV/LV ratio measurement, and ECG reading was excellent with a kappa of 0.96, 0.95, and 0.89, respectively.

**Conclusion** The algorithm had a high sensitivity of 91% and was highly reproducible. Prospective validation of the algorithm in consecutive PE patients is required before it can be used in clinical practice.

## Introduction

Chronic thromboembolic pulmonary hypertension (CTEPH) is a serious long-term complication of acute pulmonary embolism (PE). 1 In CTEPH, persistent obstruction of the pulmonary arteries causes vascular remodeling, pulmonary hypertension (PH), and right heart ventricular failure. The natural course of CTEPH includes progressive involvement of distal pulmonary arteries due to thrombotic occlusion as well as secondary vasculopathy in the nonoccluded arteries caused by redistribution of the blood flow via multiple anastomoses between the systemic and pulmonary circulation. CTEPH may be cured by pulmonary endarterectomy (PEA), 1 2 whereas patients who are deemed inoperable, due to extensive involvement of distal pulmonary arteries, have a lower survival in the first 3 years following CTEPH diagnosis (70 vs. 89%). 3 Hence, early CTEPH diagnosis is of relevance for optimal treatment and patient outcome. 2 4 5 Notably, as recently demonstrated in the European CTEPH Registry, diagnosing CTEPH at an earlier time is still a major clinical challenge with a reported median diagnostic delay of 14 months. 6 Until now international guidelines recommend to perform an echocardiography in patients with signs and symptoms suggestive of CTEPH after a PE event and do not provide clear recommendations for strategies to reduce this delay in the follow-up of patients with acute PE. 1

**Fig. 1:**
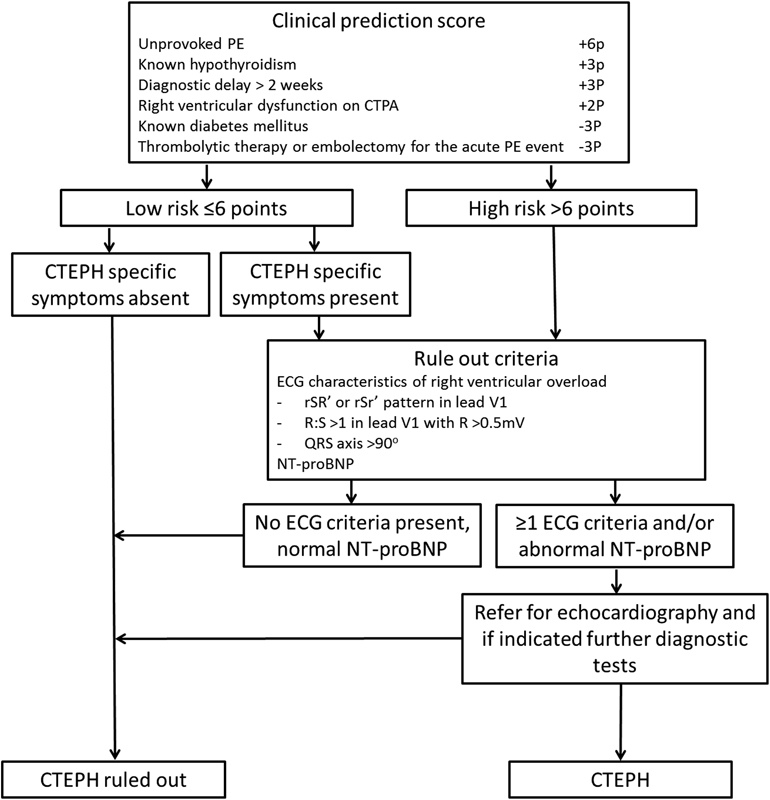
Screening algorithm for CTEPH after acute PE consisting of the CTEPH prediction score, CTEPH-specific symptoms, and the rule-out criteria. CTEPH, chronic thromboembolic pulmonary hypertension; PE, pulmonary embolism; ECG, electrocardiography; NT-proBNP, N-terminal pro-brain natriuretic peptide.

**Fig. 2:**
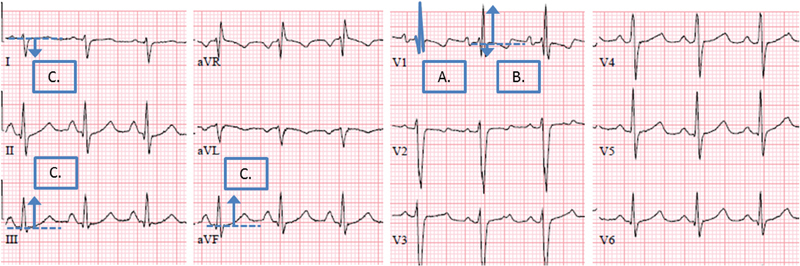
ECG demonstrating the three electrocardiographic signs of the rule-out criteria. ( **A** ) In lead V1, a right bundle branch block: rSR′ or RSr′ pattern with a QRS duration ≥ 120 ms; ( **B** ) in lead V1, R:S > 1 with R > 0.5 mV; and ( **C** ) right QRS axis deviation QRS axis >90 degrees. CTEPH, chronic thromboembolic pulmonary hypertension; ECG, electrocardiography.

Recently, a noninvasive screening algorithm for patients with a recent PE was constructed aiming at earlier CTEPH detection. This screening algorithm, consisting of sequential application of a clinical prediction score 7 and a set of rule-out criteria 8 9 within 6 months following a PE diagnosis ( Fig. 1 ), is currently being evaluated in an international multicenter prospective management study (InShape 2 study, Clinical Trials.gov identifier NCT02555137). The decision rule identifies the majority of patients with a low risk of CTEPH (i.e., six points or less) who do not need further diagnostic tests. 7 The rule-out criteria consist of electrocardiography (ECG) reading and N-terminal pro-brain natriuretic peptide (NT-proBNP) measurement with a sex- and age-dependent threshold. 8 9 These latter two tests will be applied in patients with a high pre-test probability (more than six points) or clear symptoms suggestive of CTEPH (e.g., persistence of physical impairment or dyspnea). In the absence of three specific ECG characteristics suggestive of right ventricular overload ( Fig. 2 ) and a normal age- and gender-adjusted NT-proBNP level, CTEPH is considered excluded with a sensitivity of more than 90%. 8 9 Hence, only patients with abnormal rule-out criteria need to be referred for echocardiography. 1 By this design, CTEPH diagnostic resources can be focused not only on patients with clear symptoms of CTEPH but also on those with a high pre-test probability of CTEPH, with a limited number of required echocardiographs.

Due to the relatively rare occurrence of CTEPH after acute PE (i.e., ∼3% of PE survivors), 10 the sensitivity of the algorithm can only be rigorously tested in selected patients with a much higher CTEPH prevalence. In the current study, we assessed the sensitivity of the screening algorithm in selected patients with confirmed CTEPH after acute PE to evaluate whether these patients would not have been missed by the algorithm. In addition, we assessed the reproducibility of the individual items of the algorithm.

## Methods

### Study Population

This is a retrospective analysis of consecutive patients diagnosed with CTEPH between 2014 and 2016 in the VU University Medical Center (VUMC), Amsterdam, the Dutch referral center for CTEPH. The CTEPH diagnosis was based on the results of right heart catheterization (RHC) and pulmonary angiography in all patients according to current guidelines. 1 For the present analysis, only patients with a documented previous episode of acute PE for whom the original medical charts were available were eligible for inclusion. The Institutional Review Board (IRB) of the VUMC approved the study protocol and waived the need for informed consent due to the observational nature of the study.

### Assessment of the CTEPH Screening Algorithm

All components of the CTEPH screening algorithm ( Fig. 1 ) were assessed from the original patient charts by two reviewers (Y.M.E-V. and D.R.), who were blinded for each other's findings. Using this info, the clinical prediction score 7 was calculated. A score of more than 6 points indicates a high risk of CTEPH. Furthermore, the presence of physical impairment or dyspnea in the clinical course of the index PE was evaluated by reviewing the patient charts by the same two reviewers. The ECG and NT-proBNP measurements with a sex- and age-dependent threshold performed during CTEPH diagnostic workup in the VUMC were used to apply the rule-out criteria. 8 9 ECG reading was independently performed by two reviewers as well (Y.M.E-V. and F.A.K.). For calculation of the final CTEPH prediction score and outcome of the rule-out criteria, differences were resolved by consensus.

### Study Outcome

The primary objective of the study was to evaluate the sensitivity of the screening algorithm in patients diagnosed with CTEPH, that is, the number of patients with confirmed CTEPH that would have been correctly identified according to this strategy. The secondary aim of the study was to assess the interobserver agreement for calculating the prediction score, right-to-left ventricle (RV/LV) diameter ratio measurement, and ECG reading.

### Statistical Analysis

Based on the available number of patients in the allocated time frame, a sample size of 50 patients was chosen. The sensitivity of the CTEPH screening algorithm was determined with its corresponding 95% confidence interval (CI). A sensitivity of more than 90% was predefined as adequate. Interobserver agreement for the assessment of the prediction score, RV/LV ratio measurement on CT pulmonary angiography (CTPA), as well as ECG reading was evaluated by calculating Cohen's kappa statistics. 11 The kappa value for agreement was interpreted as follows: poor (< 0.20), fair (0.21–0.40), moderate (0.41–0.60), good (0.61–0.80), or very good (0.81–1.00). All analyses were performed using SPSS software version 23 for Windows IBM Corporation.

## Results

### Patients

A total of 68 consecutive patients diagnosed with CTEPH in the period from 2014 to 2016 in the VUMC were eligible for inclusion. Of these, 14 patients were excluded because a documented previous episode of acute PE was lacking (13 patients) or detailed information of the index PE diagnosis was unavailable (one patient), leaving a total of 54 patients for the current analysis. Their baseline characteristics are shown in Table 1 . Mean age of the included patients at the time of CTEPH diagnosis was 63 ± 15 years and 26 (48%) patients were male. The mean pulmonary artery pressure (mPAP) by RHC was 42 mm Hg (±standard deviation [SD]: 12 mm Hg). Of those, 18 patients had a mPAP of less than 35 mm Hg and 11 patients had a mPAP of greater than 50 mm Hg. The median time between last PE diagnosis and CTEPH presentation was 9 months (interquartile range [IQR]: 5–15). Twenty patients were referred to the VUMC for CTEPH diagnostic workup within 6 months after the last PE diagnosis. A total of 48 patients (89%) were treated with vitamin K antagonists and 6 (11%) with direct oral anticoagulants. Twenty-two (41%) patients had a history of recurrent venous thromboembolism (VTE).

**Table 1:** Patient characteristics

	Patients ( *n* = 54)
Age at CTEPH diagnosis (y, SD)	63 (15)
Male sex ( *n* , %)	26 (48)
mPAP at diagnosis of CTEPH (average mm Hg, SD)	42 (12)
Number of VTE events (median, IQR)	1 (1–2)
Treatment of last PE	
Vitamin K antagonist ( *n* , %)	48 (89)
DOAC ( *n* , %)	6 (11)
Duration of last PE to CTEPH diagnosis (median months, IQR)	9 (5–15)
Comorbidities at the moment of CTEPH diagnostic workup	
COPD ( *n* , %)	11 (20)
Chronic left heart failure ( *n* , %)	1 (2)
Rheumatic diseases ( *n* , %)	7 (13)
Malignancy ( *n* , %)	8 (15)
Splenectomy ( *n* , %)	2 (4)
Prior infected pace maker lead ( *n* , %)	0
Known antiphospholipid syndrome ( *n* , %)	2 (4)

Abbreviations: COPD, chronic obstructive pulmonary disease; CTEPH, chronic thromboembolic pulmonary hypertension; DOAC, direct oral anticoagulants; IQR, interquartile range; mPAP, mean pulmonary artery pressure; PE, pulmonary embolism; SD, standard deviation; VTE, venous thromboembolism.

### Clinical Prediction Score

The complete prediction score could be calculated in 44 patients. In 10 patients, the clinical prediction score was incomplete, although based on the available data these patients could be indicated as low or high risk based on a definitive score of below or above 6 points. The index PE episode was unprovoked in 47 patients (87%). Three patients had known hypothyroidism at the moment of the index PE diagnosis. The diagnostic delay for the index PE was longer than 2 weeks in 45 patients. This latter information could not be retrieved for three patients. The majority of patients (44) had signs of right ventricular dysfunction as defined by a RV/LV diameter ratio of ≥ 1.0 on CTPA. Information of the RV function was not available for nine patients, of whom two had been subjected to ventilation perfusion scintigraphy to diagnose the PE. The original CTPA images could not be retrieved for the remaining seven. Five of the included patients had known diabetes mellitus and one patient received thrombolytic therapy. Based on the available data, 46 of 54 patients (85%, 95% CI: 73–93%) had a total score of at least more than 6 points indicative of high risk of CTEPH, and eight had a score of a maximum of 6 points or lower, allowing for a definite score result in all 54 patients.

Fifty patients had reported persistent dyspnea or physical impairment within the first 6 months following the index PE diagnosis. Of the eight patients with a score of 6 points or less indicating low probability, six patients had persistence of symptoms and would therefore have been subjected to the rule-out criteria according to the algorithm ( Fig. 1 ).

### Rule-Out Criteria

The rule-out criteria were evaluated in all 52 patients with either high pre-test probability or specific symptoms of CTEPH. In one of these patients, the ECG was not available. Because the NT-proBNP level was abnormal, we were able to confirm the indication for echocardiography in this patient. Of the 51 patients with an available ECG, 33 (65%) had one or more ECG criteria positive and 15 (29%) patients scored two or more ECG criteria positive. The median NT-proBNP level in all patients was 906 ng/L (IQR: 145–235,410). In 35 (67%) of the 52 patients, the NT-proBNP level was abnormal. Forty-nine patients (49/52; 94%, 95% CI: 84–99%) scored positive on at least one of the rule-out criteria.

### Sensitivity of the Screening Algorithm

According to the screening algorithm, a total of 49 out of 54 patients were correctly identified by the algorithm, implicating a sensitivity of 91% (95% CI: 79–97%). This indicates that 27 of 30 cases of CTEPH would have been detected when applying the screening algorithm to 1,000 random PE survivors with a 3% CTEPH incidence (projected negative predictive value: 99.7%; 95% CI: 99.1–99.9%).

Detailed characteristics of the five patients who were not identified by the algorithm are shown in Table 2 . Two patients with a malignancy-related provoked PE were not identified as high risk according to the clinical prediction score. Both patients developed CTEPH-specific symptoms only after a long follow-up period of 2 and 9 years after the index PE episode, respectively. The other three patients had normal ECG and NT-proBNP blood levels. Based on the diagnostic procedures performed during the CTEPH diagnostic workup, these three patients had a normal RV function and no RV dilatation at echocardiography, CTPA, and cardiac magnetic resonance imaging (MRI). Two of the three had an elevated estimated pulmonary artery pressure which was the reason for RHC. The last patient was referred for RHC because of the combination of extensive abnormalities on the ventilation perfusion scintigraphy and severe clinical symptoms ( Table 2 ).

**Table 2:** Characteristics of the five patients who were not identified according to the screening algorithm

	Patient 1	Patient 2	Patient 3	Patient 4	Patient 5
Age at CTEPH diagnosis	76	86	62	65	65
Sex	Male	Female	Male	Male	Female
NYHA classification at the time of CTEPH referral	3	4	3	2	2
Number of previous VTE events	2012: provoked PE (postsurgery, malignancy related)	1994: provoked PE, malignancy related	1999: unprovoked PE 2014: unprovoked PE	2002: unprovoked PE 2012: unprovoked DVT	2014: unprovoked PE
Referral to the VUMC (months after PE diagnosis)	23	240	6	151	6
Cardiopulmonary comorbidities	None	COPD	None	None	None
Other risk factors for CTEPH a	None	Splenectomy	None	None	Rheumatoid arthritis
Clinical prediction score	2 points	5 points	11 points	9 points	11 points
Persistence of symptoms after index PE	In 2014, new, progressive symptoms of dyspnea	In 2013, new, progressive symptoms of dyspnea	Yes	Yes	Yes
Rule-out criteria	Abnormal	Abnormal	Normal	Normal	Normal
NT-proBNP ng/L b	1,694 (<486)	9,082 (<738)	101 (<210)	56 (<376)	148 (<301)
ECG items c	1 item	2 items	None	None	None
Echocardiography (at diagnosis of CTEPH)	Dilated RV, severe PH	Dilated RV, severe PH	RV not dilated, normal function, signs of PH based on a slightly dilated right atrium and a SPAP of > 44 mm Hg	RV not dilated, normal function, signs of PH based on midsystolic notching of the pulmonary valve and a SPAP of >55 mm Hg	RV not dilated, normal function, no signs of PH. RHC performed because of severity of symptoms and the extensiveness of the abnormalities on V/Q lung scintigraphy
RHC mPAP (mm Hg)/PVR (dynes/s/cm ^5^ )	56/554	49/577	36/329	31/400	32/376

Abbreviations: CTEPH, chronic thromboembolic pulmonary hypertension; ECG, electrocardiography; mPAP, mean pulmonary artery pressure; ng/L, nanograms per liter; NT-proBNP, N-terminal pro-brain natriuretic peptide; NYHA, New York Heart Association; PE, pulmonary embolism; PH, pulmonary hypertension; PM, pacemaker; PVR, pulmonary vascular resistance; RHC, right heart catheterization; RV, right ventricle; SPAP, systolic pulmonary artery pressure; V/Q, ventilation/perfusion lung scintigraphy; VTE, venous thromboembolism; VUMC, VU University Medical Center, Amsterdam.

a Splenectomy, infected PM leads, autoimmune diseases.

b Age and sex adjusted.

c Right bundle branch block: rSR′ or RSr′ pattern in lead V1 with a QRS duration ≥ 120 ms, R:S > 1 in lead V1 with R > 0.5 mV or right QRS axis deviation QRS axis > 90 degrees.

### Interobserver Variability

The Cohen kappa statistic between the two reviewers was 0.96 for calculating the prediction score, 0.95 for measuring the RV/LV diameter ratio based on a ratio of <1 or ≥1, and 0.89 for ECG reading.

## Discussion

With this study we could demonstrate that by using a simple noninvasive CTEPH screening algorithm, 49 out of 54 CTEPH patients could have been correctly identified early after the PE diagnosis. The sensitivity of the screening algorithm in this population was thus 91% (95% CI: 79–97%). The screening algorithm proved highly reproducible as well, with Cohen's kappa-statistics of 0.96, 0.95, and 0.89 for calculating the prediction score, RV/LV ratio measurement, and ECG reading, respectively.

Early CTEPH diagnosis is of relevance for optimal treatment and outcome of patients suffering from this disease. Although randomized trials comparing early and later CTEPH diagnosis and treatment initiation are not available, it is reported in the European registry that performing PEA was the strongest predictor of survival (hazard ratio: 0.37; 95% CI: 0.24–0.58; *p* < 0.0001) underlining the importance of early CTEPH diagnosis. 3 Until now, however, strategies for earlier CTEPH diagnosis are rarely reported in the literature and are underreported in relevant guidelines. 12 The median time between last reported PE event and referral for CTEPH diagnostic workup in this cohort was 9 months.

Based on the screening algorithm evaluated in this analysis, only 5 of 54 CTEPH patients would have been missed. Two of these patients suffered provoked PE many years before the CTEPH diagnosis, and had full physical recovery before new symptoms suggestive of CTEPH occurred. A recent study suggested that CTEPH often is an already ongoing disease in patients diagnosed with acute PE. 13 In this study, it was shown that five of seven CTEPH patients from 146 patients with PE already had signs of CTEPH at echocardiography during initial PE diagnosis and all seven had signs of CTEPH on retrospective CTPA evaluation. Our two patients may either have developed a secondary vasculopathy caused by redistribution of the blood flow after PE with a long symptom-free honeymoon period or developed subclinical recurrent PE as start of developing CTEPH. 14 15

The three other patients who were not identified by the algorithm had a high risk according to the CTEPH prediction score, displayed characteristic symptoms of CTEPH, but had normal ECG and NT-proBNP levels. Interestingly, echocardiography, CTPA, and cardiac MRI performed during CTEPH diagnostic workup showed a normal RV function and no RV dilation in all three patients. This may be explained by the process of RV adaptation to the increased vascular load. 16 During this stage of PH which is also referred to as “coupling,” the right ventricle adapts by increasing contractility and muscle wall thickness to maintain flow. 17 In the natural course of disease, “uncoupling” will ultimately occur, causing RV dilatation and eventually RV failure. The fact that two of the three patients were referred within 6 months after the PE diagnosis suggests that these patients were indeed identified early in the course of disease. Considering this, we conclude that patients in very early stages of CTEPH may be missed by the rule-out criteria, as was shown in the derivation study of the criteria. Even so, the majority (18/20) of patients referred within the first 6 months after PE diagnosis and most (16/18) patients with mild increased mPAP (<35 mm Hg) were correctly identified by the algorithm.

The strength of this study lies in the large cohort of consecutive patients diagnosed with CTEPH after a previously documented episode of acute PE, as well as the ability to assess the interobserver variability of all individual items of the screening algorithm.

This study also had some limitations. The design of this study does not allow us to estimate the specificity of the algorithm. Also, only limited data with regard to index PE event of patients referred to VUMC before 2014 were available. Therefore, we were not able to include more patients in this study. Based on the available data from the referral centers, it was not possible to evaluate the complete screening algorithm in all CTEPH patients. We were nonetheless able to include 10 patients with an incomplete clinical prediction score with definitely more than 6 or definitely less than 6 points. The patient with a missing ECG had an abnormal NT-proBNP level, allowing for full assessment of the rule-out criteria in all patients. Another limitation is that the median time between the last PE diagnosis and referral to the VUMC for CTEPH diagnostic workup was 9 months. We used ECGs and NT-proBNP measurements at the time of referral and not the required 3 to 6 months following acute PE, which could have influenced our outcome. Lastly, although the screening algorithm is assessed for the early diagnosis of CTEPH, the sensitivity was tested in prevalent patients, some with advanced disease.

In conclusion, 91% of the evaluated CTEPH patients would have been identified by the proposed screening algorithm, underlining its adequate sensitivity. All components of the algorithm proved to be highly reproducible as well. The few patients who would have been missed by the algorithm had either a very long “honeymoon period” or were diagnosed with very early disease. The results of the ongoing prospective validation of the algorithm in consecutive PE patients will provide more definite proof of sensitivity and also the accuracy and applicability of the algorithm in daily clinical practice.
